# Research on Colorization Algorithm for γ-Photon Flow Field Images Using the SECN Model

**DOI:** 10.3390/e28040414

**Published:** 2026-04-04

**Authors:** Hui Xiao, Liying Hou, Jiantang Liu, Shengjun Huang

**Affiliations:** 1 College of Automation Engineering, Nanjing University of Aeronautics and Astronautics, 29 Jiangjun Road, Nanjing 211106, China; liyinghou72@163.com (L.H.);; 2 Shenzhen Research Institute, Nanjing University of Aeronautics and Astronautics, Shenzhen 518110, China; 3 College of Computer Science and Technology, Nanjing University of Aeronautics and Astronautics, 29 Jiangjun Road, Nanjing 211106, China

**Keywords:** photon imaging, flow field monitoring, SECN model, image colorization

## Abstract

γ-photon tomography, which leverages the high penetration and electrical neutrality of high-energy γ-photons, offers a promising non-contact approach for industrial flow field monitoring. However, γ-photon flow-field images are inherently grayscale and exhibit probabilistic statistical imaging characteristics, leading to color banding artifacts when processed by mainstream colorization algorithms like DeOldify, which compromise structural continuity and visual consistency. To address this issue, this paper proposes a Structure Enhancement Colorization Network (SECN) model for γ-photon flow-field image colorization. A U-Net + GAN framework is employed, with ResNet101 as the generator backbone. It integrates structure-aware enhancement and multi-scale attention modules, while the discriminator incorporates enhanced blocks for improved boundary and texture discrimination. By adaptively fusing global–local features across channel and spatial dimensions, the SECN model effectively suppresses color banding artifacts and enhances structural consistency. To validate the effectiveness of the proposed algorithm, two CFD-simulated γ-photon flow-field image colorization scenarios—namely a large-scale vortex wake and a horizontal wake—are used as evaluation targets. In terms of image quality metrics, the proposed colorization algorithm achieves PSNR, SSIM, FID, and MAE values of 32.5831, 0.8612, 17.8514, and 0.0191, respectively, corresponding to improvements over DeOldify of 4.54%, 2.82%, 5.18%, and 11.16%. When considering information entropy, the proposed colorization algorithm achieves an average entropy value of 4.0257, marking a 4.44% increase compared to DeOldify’s 3.8543, demonstrating superior information preservation and reduced uncertainty in reconstructing complex probabilistic structures. Furthermore, from the perspective of parameter inversion, the temperature inversion MAPE is 7.60%, which is a significant reduction of 18.42% compared to that of DeOldify.

## 1. Introduction

When a radionuclide undergoes β^+^ decay, a positron is generated. This positron swiftly (within approximately 10^−12^ s) interacts with a nearby electron, resulting in an annihilation event. During this process, a pair of γ photons, each with an energy of 511 keV, are emitted in nearly opposite (180°) directions. Each photon pair defines a line of response (LOR), which contains information such as the photon time-of-flight, energy, and spatial location. An external detection system captures LOR data by applying energy and timing windows; the recorded LORs are termed coincidence events. Subsequently, computerized image reconstruction techniques are employed to estimate the annihilation location along the LOR. By statistically counting the number of annihilation events and normalizing them into image pixels, γ-photon tomographic imaging is achieved. Owing to their strong penetration and electrical neutrality, high-energy γ photons enable a detection process that is theoretically resilient to harsh conditions, including high temperature, high pressure, and electromagnetic interference. This makes γ-photon tomography a promising non-contact solution for industrial flow-field monitoring [1,2].

However, γ-photon flow-field images are grayscale, with visual information confined to a single intensity dimension [3]. To facilitate industrial condition monitoring, it is essential to convert these grayscale images into color images. The DeOldify algorithm has emerged as a mainstream approach for grayscale image colorization, thanks to its well-designed U-Net generator architecture and stable adversarial training framework, which deliver impressive visual quality in natural image colorization. Nevertheless, the original U-Net structure may still exhibit limitations such as insufficient color saturation, blurred details, and color inconsistency when processing images with rich details or strong logical structures. To mitigate dull-color and artifact-prone outputs, Oliverio and Prasetyo proposed a Modified Multi-Attention U-Net [4], which adaptively focuses on key content regions and important color-feature channels, producing more vivid and sharper colorized results in complex scenarios, such as historical black-and-white photographs. For tasks requiring precise attention to target structures to ensure consistent outputs, Schlemper et al. introduced Attention Gated Networks [5]. These networks incorporate attention mechanisms into the skip connections of U-Net, enabling the model to emphasize critical target regions while suppressing irrelevant background responses, thereby providing valuable insights for improving regional color consistency. Yet, γ-photon image reconstruction is inherently a probabilistic statistical imaging process. It assumes that each response line is generated according to the probability contributions (defined by the system matrix) of the pixels traversed by the LOR. Consequently, in the reconstructed γ-photon image, the annihilation location is represented as a Gaussian-like distributed pixel region centered around the true annihilation point, while the actual annihilation event occurs at a specific spatial coordinate. As a result, the sharp edges present in the real flow field are smoothed into continuous probability density gradients in the reconstructed grayscale images. This blurring is governed by the system’s inherent Gaussian-like point spread function, which arises from Poisson-distributed coincidence counts and the statistical nature of the OSEM reconstruction. When mainstream colorization algorithms like DeOldify are applied, a fundamental structural mismatch occurs. These CNN-based models, trained on natural images, assume strong local pixel correlations and smooth color transitions. However, the slow and spatially varying probability gradients in γ-photon images violate this assumption. The localized receptive fields interpret gradual density variations as abrupt semantic or illumination changes, implicitly performing feature-space thresholding. Consequently, the decoder quantizes continuous distributions into discrete color steps, producing stair-like transitions known as color banding artifacts. In essence, the probabilistic imaging process renders conventional natural-image colorization networks fundamentally ill-suited for recovering the smooth gradients present in γ-photon flow-field images. From an information-theoretic perspective, this probabilistic nature of γ-photon imaging introduces inherent uncertainty in the reconstructed images, where grayscale intensity distributions reflect varying levels of information content and randomness. Shannon entropy quantifies the average uncertainty or informational richness in an image by measuring the expected information needed to describe the pixel intensity distribution [6,7]. In image restoration and colorization tasks, higher entropy in the restored image typically indicates better preservation of structural details and reduced information loss, while artifacts like banding or over-smoothing can decrease entropy by introducing predictability or redundancy [8]. This motivates the use of entropy concepts to evaluate colorization quality in γ-photon flow-field images, as the task involves reconstructing complex probabilistic structures with minimal uncertainty. To address these challenges, this paper proposes a γ-photon flow-field image colorization algorithm based on the SECN model. A U-Net + GAN framework is constructed, where the generator adopts ResNet101 as the backbone and incorporates structure-aware enhancement and multi-scale attention modules. The discriminator integrates enhanced blocks to strengthen boundary and texture discrimination and adaptively fuses global–local features across channel and spatial dimensions, thereby suppressing color banding artifacts and improving structural consistency.

## 2. Relevant Theory

The primary challenge in image color restoration lies in recovering visually realistic and semantically consistent color information from images lacking chromatic cues. The performance of this process hinges on precise feature extraction and effective modeling of the color-mapping relationship. This section systematically reviews the core theories and key techniques pertinent to γ-photon flow-field grayscale image colorization, including the fundamental principles of CNNs (convolutional neural networks) for image feature extraction and the application mechanisms of U-Net and GANs (generative adversarial networks) in image colorization, thereby providing theoretical underpinnings for subsequent model design.

### 2.1. Image Feature Extraction Algorithms Using Convolutional Neural Networks

CNNs are pivotal in driving deep learning advancements in computer vision [9]. Their key strength lies in the automatic and efficient extraction of hierarchical image features, ranging from low-level to high-level, by mimicking the layered organization of the biological visual cortex [10]. A typical CNN comprises convolutional layers, pooling layers, and fully connected layers. Convolutional layers utilize convolutional kernels (filters) to perform sliding window operations on input images, detecting local features such as edges, corners, and textures [11]. Pooling layers, such as max pooling or average pooling, down-sample feature maps to reduce spatial resolution, thereby decreasing the number of parameters and computational cost while conferring a degree of translation invariance to the model [12].

However, conventional CNNs, constrained by their inherently local receptive fields, face limitations in capturing long-range dependencies [13]. To address this, attention mechanisms have been integrated into CNN architectures [14]. These mechanisms enable models to dynamically assign varying weights to different spatial locations or feature channels, thereby focusing on the most relevant information for the task at hand. Building on this, self-attention mechanisms from Transformer architectures have been adapted for visual tasks, leading to models like Vision Transformer (ViT) [15] and Swin Transformer [16]. These Transformer-based models efficiently capture long-range dependencies in images through self-attention, gathering global contextual information crucial for understanding complex scenes. Such advanced feature extraction paradigms provide robust semantic and structural foundations for downstream image generation and restoration tasks, including image colorization.

In image color restoration, the feature extraction capability of CNNs directly influences the accuracy of color mapping. Shallow features retain edge and texture information, constraining the detail fidelity of color restoration and preventing edge blurring or color bleeding. Conversely, high-level semantic features guide reasonable color assignment through category-aware representations, thereby reducing semantic inconsistency. Therefore, CNN-based feature extraction algorithms form a cornerstone of constructing high-performance color restoration models, with their hierarchical representations offering comprehensive support from pixel-level details to image-level semantics.

### 2.2. Image Colorization Algorithm Using U-Net and GAN Networks

Image colorization aims to restore natural and realistic color information from grayscale images (or low-quality color images). The integration of U-Net, which excels in preserving fine details, with GAN-based adversarial learning, which promotes realistic image generation, has emerged as a mainstream technical approach, significantly enhancing colorization quality. U-Net performs admirably in image generation and restoration tasks due to its encoder–decoder architecture and skip connections. The encoder progressively down-samples the input to extract multi-scale contextual features, while the decoder gradually restores spatial resolution through up-sampling. The innovation of skip connections lies in directly forwarding shallow, high-resolution features from different encoder stages to the corresponding decoder stages, enabling effective fusion of deep semantic information with low-level details [17]. This design mitigates the loss of spatial information in deep networks, which is crucial for colorization tasks requiring accurate pixel-level predictions [18]. Consequently, many modern colorization models adopt U-Net or its variants as the generator backbone, producing detail-rich images with precise spatial structures [19].

Despite U-Net’s strong structural fidelity, training solely with pixel-wise loss functions (e.g., L1 or L2) is often limited by the regression-to-the-mean effect. Image colorization is inherently multimodal: a single grayscale input can correspond to multiple plausible chrominance distributions. Pixel-wise objectives tend to minimize statistical error by predicting the mean of this distribution, leading to desaturated colors and loss of high-frequency texture details [20]. GAN-based adversarial learning offers an effective solution by introducing an adversarial loss [21]. GANs consist of a Generator and a Discriminator that compete during training [22]. In colorization tasks, the Generator (typically employing a U-Net-like architecture) colors grayscale images, while the Discriminator learns to distinguish between images “painted” by the Generator and real color images [23]. This adversarial loss encourages the Generator to learn the data distribution of authentic images through the competitive game with the Discriminator, resulting in more realistic images visually indistinguishable from genuine ones [24]. The Pix2Pix model exemplifies a typical application of conditional GANs (cGANs) in image translation tasks, demonstrating that combining adversarial loss with traditional L1/L2 loss can produce high-quality colorized images.

Most mainstream image colorization methods adopt a hybrid architecture, utilizing U-Net as the generator and employing GAN-based adversarial training [25]. This combination leverages U-Net’s ability to preserve spatial details and the Discriminator’s capacity to enhance perceptual realism. For instance, ChromaGAN follows this hybrid design and further incorporates semantic information to guide the colorization process. However, adversarial training can be unstable and may suffer from mode collapse [26]. To address this, He et al. integrated VAE with GAN to enhance sample diversity and stabilize training. Additionally, emerging paradigms such as self-supervised learning and meta-learning have been explored to reduce reliance on large-scale paired data and improve generalization across diverse scenarios by designing proxy tasks or learning adaptive loss functions [27]. These advancements offer valuable insights for colorization on specialized data, such as γ-photon flow-field images.

## 3. SECN-Based Colorization Algorithm for γ-Photon Flow Field Images

### 3.1. SECN Model

The SECN framework is mainly constructed around the cGAN paradigm. This approach involves training a Generator and a Discriminator in a dynamic adversarial process to achieve high-quality color mapping from single-channel grayscale γ-photon flow-field images. The Generator’s objective is to synthesize realistic three-channel color images that accurately replicate the subtle color distributions and textural details inherent in the underlying flow field. To mitigate color banding artifacts and enhance structural continuity, the conventional GAN architecture has been customized. Specifically, the Generator employs a robust U-Net architecture with a ResNet101 backbone, where skip connections effectively fuse deep semantic information with high-frequency details. Notably, the proposed Structure-Aware Enhancement (SAE) module is incorporated into both the Generator and Discriminator. SAE modules are embedded at multiple decoder stages in the Generator to facilitate smooth and continuous color transitions. On the Discriminator side, the SAE module bolsters the detection of staircase-like discontinuities and provides the Generator with structure-informative gradient feedback.

**Figure 1:**
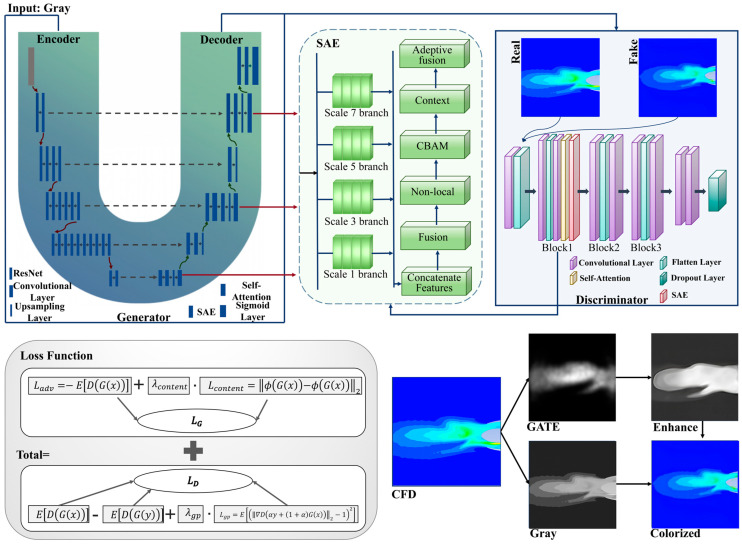
Overall Architecture of the SECN Model.

The entire SECN model undergoes end-to-end optimization using a hybrid loss function that combines an adversarial component (based on the Wasserstein distance) and a content component (based on VGG perceptual features). During training, the Discriminator compares the generated image against the ground truth, providing adversarial feedback. Simultaneously, the content loss measures the discrepancy between the generated and real images in the feature space, ensuring structural and semantic consistency. The total loss function jointly updates the parameters of both networks through backpropagation. Through this closed-loop optimization process of “generation–discrimination–structure enhancement”, the SECN effectively reduces cross-region color discontinuities and produces high-fidelity colorized images with improved structural consistency, as demonstrated in Figure 1.

### 3.2. Generator

**Figure 2:**
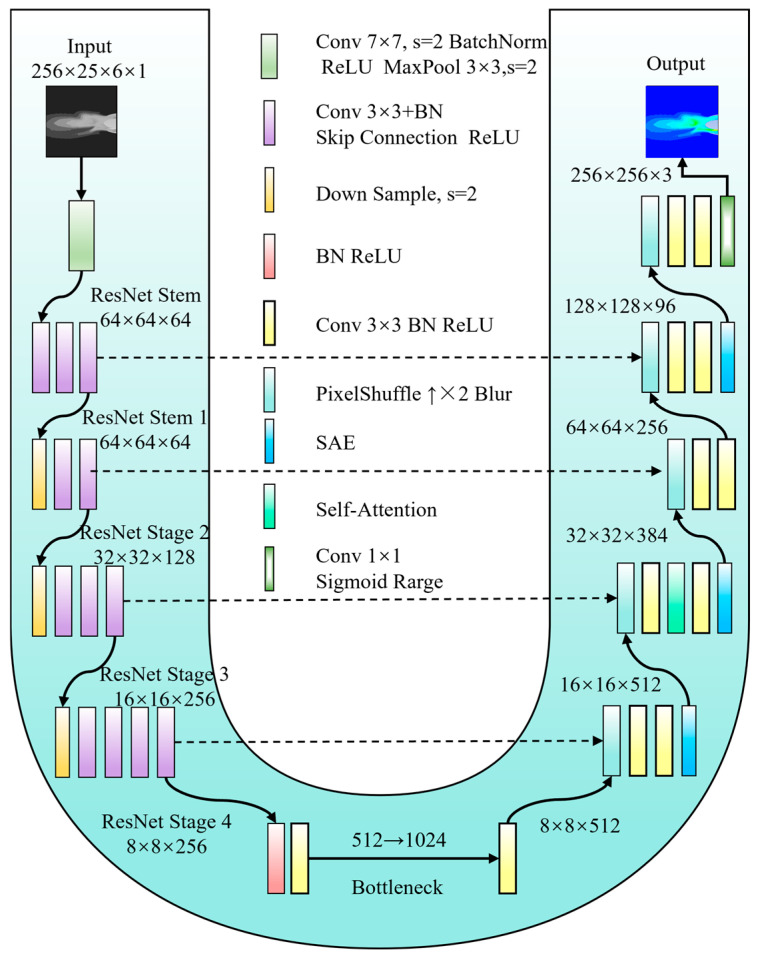
Detailed Architecture of the Generator Network.

To achieve a harmonious balance between robust feature representation and high-quality image reconstruction, the generator employs a symmetric encoder–decoder architecture modeled after U-Net. The encoder leverages a ResNet101 backbone, which has been pre-trained on the ImageNet dataset, and this enables it to perform hierarchical feature extraction, capturing both low-level textures and high-level semantics within flow-field images. In the decoding phase, up-sampling is carried out efficiently through PixelShuffle operations, initialized using the ICNR method. This process progressively restores the spatial resolution of the image. At the junction between the encoder and decoder, two cascaded convolutional blocks are introduced. These blocks are designed to explicitly enhance cross-channel interactions by expanding and then compressing the number of channels (ni→2ni→ni). This refinement process focuses on the core semantic information that is most pertinent to colorization. In addition, the classic U-Net skip connections are preserved in the architecture. These connections concatenate high-resolution features from the encoder with their corresponding decoder stages, effectively fusing deep semantics with shallow details. This fusion mechanism plays a crucial role in preventing the loss of critical structural features, as illustrated in Figure 2.

The generator’s key innovation lies in the integration of the SAE module as a plug-and-play structural constraint unit. This module is strategically positioned at three decoder stages: low, middle, and high resolutions. At the low stage (high resolution), where the network primarily focuses on reconstructing local details, the SAE module enhances edge and texture continuity, effectively preventing edge blurring. Moving to the middle stage, the SAE module facilitates smooth color transitions within regions and their adjacent boundaries, thereby minimizing spatial discontinuities. At the high stage (low resolution), where the overall tone is established, the SAE module leverages long-range dependency modeling to enforce global semantic tonal consistency and prevent color drift across different regions. Through this layered and targeted structure enhancement approach, the generator is guided by multi-scale spatial cues throughout the reconstruction process. This fundamentally suppresses color banding artifacts and ensures structurally coherent transitions in the generated images.

### 3.3. Discriminator

**Figure 3:**
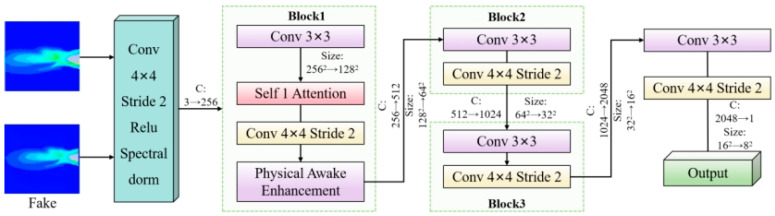
Detailed Architecture of the Discriminator Network.

The discriminator adopts a multi-scale deep convolutional architecture to rigorously evaluate image authenticity across various feature levels. It consists of stacked convolutional blocks, each of which extracts features through convolutional layers followed by strided convolutions for down-sampling. This design progressively reduces the feature map resolution while expanding the receptive field, enabling a comprehensive assessment ranging from local textures to global spatial layouts. To stabilize adversarial training, spectral normalization is applied to all convolutional layers, ensuring that the spectral norm of the weight matrices adheres to the 1-Lipschitz continuity requirement for WGAN-GP. A distinguishing feature of the SECN discriminator is its structure-aware discrimination capability. Unlike standard discriminators that treat all pixels uniformly, an SAE module is inserted after the first convolutional block to encourage early-stage features to focus on structure-sensitive regions, such as jet boundaries and areas with smooth gradient transitions. Consequently, when the generator produces images with unnatural color banding artifacts, the structure-aware discriminator can more effectively detect these discontinuities and deliver stronger penalizing gradients. This drives the generator to produce visually realistic and structurally continuous outputs, as illustrated in Figure 3.

### 3.4. SAE Module

**Figure 4:**
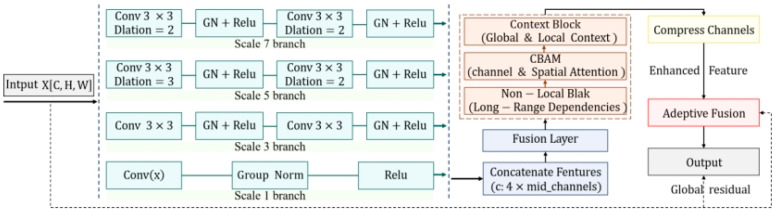
Detailed Architecture of the SAE Module.

The SAE module stands as the pivotal technical innovation in this study, acting as the “engine” that endows the SECN with structure-reasoning capabilities. Given an input feature map, the SAE module processes it through a sequential pipeline of construction, association, and refinement. Initially, it constructs a discriminative feature foundation by leveraging a multi-scale dilated convolutional network, which captures features at varying scales. Subsequently, it establishes global dependencies across the feature map using a NonLocalBlock, thereby enhancing the model’s awareness of long-range contextual relationships. Finally, the module refines and re-weights these features through the CBAM, which integrates both channel and spatial attention mechanisms. The result is an enhanced feature map that integrates multi-scale details, long-range dependencies, dual-attention weights, and contextual priors, as illustrated in Figure 4.

Upon entering the SAE module, feature maps are initially processed by a multi-scale dilated convolutional fusion network comprising four parallel branches. Branch 1 uses a 1 × 1 convolution to reduce dimensionality and facilitate inter-channel interaction with minimal computational cost. Branch 3 employs two cascaded 3 × 3 convolutions, yielding an equivalent receptive field of 5 × 5, to capture medium-scale local features. Branch 5 uses a 3 × 3 convolution with a dilation rate of 2, achieving an effective receptive field of 5 × 5, thereby capturing broader spatial context without increasing the number of parameters. Branch 7 adopts a 3 × 3 convolution with a dilation rate of 3, resulting in an effective receptive field of 7 × 7, to capture the most critical global structural cues. Compared to three- or five-branch designs, the four-branch scheme strikes a better balance among accuracy, convergence speed, and computational efficiency, providing a stable and discriminative foundation for subsequent attention mechanisms. The feature maps from the four branches are concatenated along the channel dimension to form a multi-scale feature representation, as described by Formula (1).

(1) $$F_{\mathrm{multi}}=\left[F_{1};F_{3};F_{5};F_{7}\right],$$

where $F_{1}$, $F_{3}$, $F_{5}$, and $F_{7}$ represent the output features of the four branches, respectively, and $\left[\cdot ;\cdot \right]$ denotes channel-wise concatenation. The concatenated feature map then undergoes feature fusion and smoothing through a 3 × 3 convolution, resulting in the fused feature $F_{\mathrm{fused}}$, as shown in Formula (2):

(2) $$F_{\mathrm{fused}}=Conv3\times 3(F_{\mathrm{multi}}),$$

The fusion operation learns the correlations among features at different scales, producing a richer and more robust representation.

To enhance training stability and feature quality, each convolutional layer is followed by GroupNorm normalization and a ReLU activation function. Compared to BatchNorm, GroupNorm generally offers better generalization, particularly in small-batch training scenarios.

**Figure 5:**
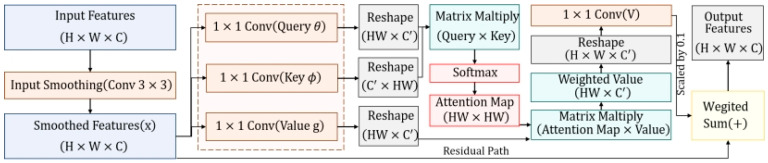
Architecture of the NonLocalBlock Module.

Subsequently, the features are fed into a non-local attention module (NonLocalBlock) to establish long-range dependencies between any two pixels in the feature map. Unlike local convolutions, the NonLocalBlock captures global information in a single step by computing a global similarity matrix and performing attention-weighted aggregation. Specifically, it calculates the similarity between each position and all positions to form a global attention map, which is then used as weights to aggregate features across all positions. The aggregated global information is transformed and added to the smoothed input features via a scaled residual connection. The output response of the entire module at location *i*, denoted as *Z_nonlocal_*, can be expressed using Formula (3):

(3) $$Z_{\mathrm{nonlocal},i}=\alpha \cdot W\left(\sum_{\forall j}\frac{\exp(\theta {\left(F_{\mathrm{fused},i}^{\prime }\right)}^{T}\phi (F_{\mathrm{fused},j}^{\prime }))}{\sum_{\forall k}\exp(\theta {\left(F_{\mathrm{fused},i}^{\prime }\right)}^{T}\phi (F_{\mathrm{fused},k}^{\prime }))}g(F_{\mathrm{fused},j}^{\prime })\right)+F_{\mathrm{fused},i}^{\prime },$$

where θ, ϕ, and g denote 1 × 1 convolutions for feature projection, W represents a 1 × 1 convolution for information fusion, and α is the scaling factor. The end-to-end design ensures that each output pixel incorporates global information from the entire feature map, maintaining high training stability while capturing long-range physical relationships. The structural diagram is shown in Figure 5.

**Figure 6:**
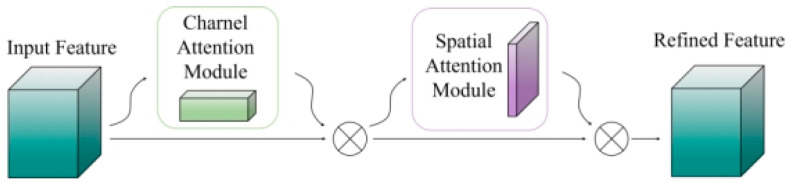
Structure of the CBAM Attention Mechanism.

Subsequently, the feature map is processed by a Convolutional Block Attention Module (CBAM), which employs a self-attention mechanism with dual attention pathways, namely channel attention and spatial attention, to accurately pinpoint and enhance key feature regions [28]. The CBAM attention structure is depicted in Figure 6.

**Figure 7:**
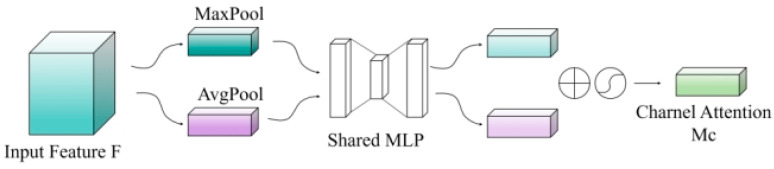
Structure of the Channel Attention Submodule.

The channel attention mechanism is designed to determine the significance weights of different channels within the feature maps. By adaptively adjusting feature responses across channels, it accentuates those channels most relevant to the image coloring task. First, the input feature map $X\in R^{C\times H\times W}$ undergoes both global average pooling (GAP) and global max pooling operations, yielding two channel descriptors, as detailed in Formulas (4)–(6):

(4) $$F_{\mathrm{avg}}=F_{\mathrm{avg}}^{c}(X)=\frac{1}{H\times W}\times \sum_{i=1}^{H}\sum_{j=1}^{W}X_{c}(i,j),$$

where $F_{\mathrm{avg}}$ represents the average pooling result, *C* denotes the number of channels, and *H* as well as *W* are the height and width of the feature map, respectively.

(5) $$F_{\max}=F_{\max}^{c}(X)=\max_{i,j}X_{c}(i,j),$$

(6) $$M_{c}(X)=\sigma \left(MLP(F_{\mathrm{avg}})+MLP(F_{\max})\right),$$

where $F_{\max}$ denotes the max-pooling result. Subsequently, these two descriptors are processed through a shared multi-layer perceptron (MLP) to derive the channel attention weights:

Here, σ represents the Sigmoid activation function. The MLP consists of two fully connected layers, with an intermediate layer having C/r channels, where r is the dimension reduction ratio (set to 16 in this study). To enhance training stability, GroupNorm normalization and ReLU activation functions are applied after each fully connected layer. The channel attention structure is illustrated in Figure 7.

The spatial attention mechanism focuses on the importance of various spatial locations within the feature maps by generating spatial attention maps that highlight the most critical regions for image coloring. First, average pooling is applied along the channel dimension, as shown in Formula (7).

(7) $$F_{\mathrm{avg}}^{s}=\frac{1}{C}\times \sum_{c=1}^{C}X_{c}(i,j),$$

This is followed by max pooling to produce two spatial descriptors, as indicated in Formula (8):

(8) $$F_{\max}^{s}=\max_{c}X_{c}(i,j),$$

These descriptors are then concatenated along the channel dimension and passed through a 7 × 7 convolutional kernel to capture spatial correlations, as described in Formula (9).

(9) $$M_{s}(X)=\sigma \left(f^{7\times 7}\left(\left[F_{\mathrm{avg}}^{s};F_{\max}^{s}\right]\right)\right),$$

where [·;·] represents channel dimension concatenation, and σ denotes the Sigmoid activation function. GroupNorm normalization is applied after the convolution to further enhance training stability.

**Figure 8:**
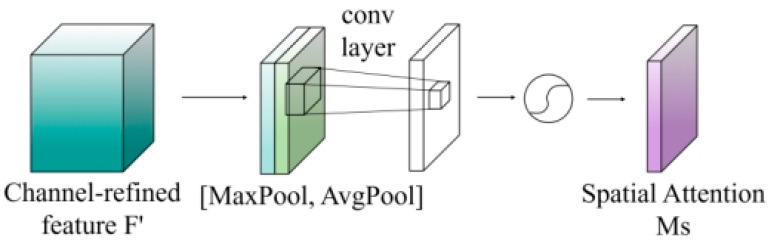
Structure of the Spatial Attention Submodule.

The CBAM integrates both channel attention and spatial attention mechanisms to form a dual attention framework. The complete computation process is articulated in Formula (10):

(10) $$X^{\prime }=M_{c}(X)⊗X,$$

where ⊗ denotes element-wise multiplication and $X^{\prime }$ represents the feature map after channel attention processing, as shown in Formula (11).

(11) $$X^{\prime \prime }=M_{s}(X^{\prime })⊗X^{\prime },$$

where $X^{\prime \prime }$ represents the feature map after spatial attention processing. The spatial attention structure is illustrated in Figure 8.

This dual-attention design aims to highlight key physical regions in images, such as temperature cores or jet boundaries. By integrating the CBAM, the model gains the capability to adaptively discern “which features” are significant and “where they matter most”.

After attention weighting, the feature map undergoes further refinement within a context-aware module (ContextBlock). This module adeptly fuses global priors with local details by capturing global contextual statistics through GAP while simultaneously preserving spatial details through local convolutions. By combining these two components, it generates adaptive weights for each pixel’s color decision, striking a balance between overall scene context and local neighborhood information. Consequently, color choices are more harmonious with the global environment. The resultant high-dimensional features are then channeled into a progressive feature compression module for dimensionality reduction. This module employs two consecutive 1 × 1 convolutions to eliminate redundant information while retaining and condensing core color-related features.

Finally, an adaptive fusion module (Adaptive Fusion) dynamically determines the fusion ratio between the enhanced features and the original features. The fused features are combined with the original input features through a weighted residual connection, with the original-branch weight coefficient set to 0.1. This ensures that the network can learn complex transformations while preserving the fundamental structure and luminance information of the input image. From an information-theoretic perspective, the SAE module optimizes information flow within the feature map. The multi-scale dilated convolutions and NonLocalBlock capture diverse spatial dependencies, thereby reducing local uncertainty. Meanwhile, CBAM’s dual-attention mechanism adaptively re-weights channels and spatial locations based on feature relevance, effectively minimizing redundant information and preserving high-entropy structural details that are crucial for γ-photon flow-field reconstruction. This design adheres to principles wherein attention mechanisms guide models towards lower-entropy representations of irrelevant features and higher retention of task-relevant information. Through this multi-stage enhancement process, the SAE module enforces geometric continuity and suppresses color banding artifacts, resulting in images with high-fidelity gradient distributions. This provides a robust foundation for quantitative analysis and supports enhanced accuracy in downstream flow-field temperature inversion tasks.

### 3.5. Loss Functions

The loss function for the generator comprises two primary components: adversarial loss and content loss. Conversely, the discriminator’s loss function is designed to enhance its ability to distinguish between real and generated images.

The total loss function for the generator is defined in Formula (12):

(12) $$L_{G}=L_{\mathrm{adv}}+\lambda_{\mathrm{content}}\times L_{\mathrm{content}},$$

where $L_{\mathrm{adv}}$ represents the adversarial loss, $L_{\mathrm{content}}$ denotes the content loss, and $\lambda_{\mathrm{content}}$ is the weighting coefficient for the content loss.

The adversarial loss uses the Wasserstein distance, as calculated in Formula (13):

(13) $$L_{\mathrm{adv}}=-E[D(G(x))],$$

where G(x) denotes the image generated by the generator, D(·) represents the output of the discriminator, and E [·] denotes the expectation.

The content loss uses feature loss, where features are extracted with a pre-trained VGG network to measure the distance between generated and real images in feature space, as shown in Formula (14).

(14) $$L_{\mathrm{content}}=||\phi (G(x))-\phi (y)|{|}_{2},$$

where φ(·) denotes the intermediate layer feature extractor of the VGG network, y represents the ground truth color image, and $||\cdot |{|}_{2}$ denotes the L2 norm. Feature loss ensures the preservation of structural consistency and semantic information in the generated images.

The discriminator’s loss function is based on the Wasserstein loss, as depicted in Formula (15):

(15) $$L_{D}=E[D(G(x))]-E[D(y)]+\lambda_{\mathrm{gp}}\times L_{\mathrm{gp}},$$

where E[D(G(x))] denotes the expected discriminator score for the generated image, E[D(y)] denotes the expected discriminator score for the real image, $L_{\mathrm{gp}}$ is the gradient penalty term, and $\lambda_{\mathrm{gp}}$ is the gradient penalty weight (set to 10 in this study).

The gradient penalty term, which aids in stabilizing the training process, is computed as shown in Formula (16):

(16) $$L_{\mathrm{gp}}=E\left[{\left(||\nabla D(\alpha y+(1-\alpha )G(x))|{|}_{2}-1\right)}^{2}\right],$$

The content loss weight $\lambda_{\mathrm{content}}$ is determined using an adaptive weight adjustment strategy. This strategy dynamically modifies the weight based on the training progress, as described in Formula (17):

(17) $$\lambda_{\mathrm{content}}(t)=\lambda_{{\mathrm{content}}_{\mathrm{base}}}\times \left(1+\alpha \times \left(1-\frac{t}{T}\right)\right),$$

where t represents the current training step, T is the total number of training steps, and α is the adjustment coefficient (set to 0.2). During the initial training phase, a higher content loss weight is employed to maintain structural integrity. As training progresses, the weight gradually decreases to enhance the effects of adversarial training. This adaptive strategy effectively balances the requirements of different training stages, preventing structural distortion early on and ensuring sufficient color saturation in later stages.

The selection of the gradient penalty weight $\lambda_{\mathrm{gp}}$ primarily considers the theoretical requirements of Wasserstein GANs (WGANs). WGANs necessitate that the discriminator satisfies the 1-Lipschitz constraint, which the gradient penalty term enforces to ensure training stability. The value range for this term is approximately [0, 1], necessitating appropriate weighting to effectively contribute to the total loss. A value of $\lambda_{\mathrm{gp}}$ = 10, as recommended in the WGAN-GP paper and validated across multiple datasets, is adopted as an empirical value [29].

## 4. Experimental Validation

### 4.1. Experimental Platform

#### 4.1.1. Data Simulation Platform

**Figure 9:**
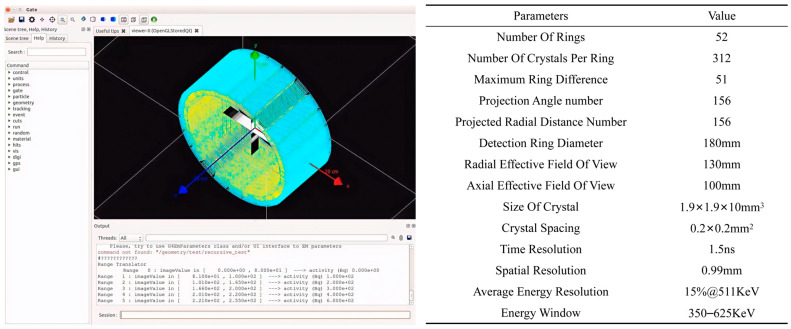
γ-photon Detection System and Key Parameters.

To acquire high-fidelity image data that accurately captures the physical characteristics of the internal engine flow field, this study employs professional CFD software, ANSYS (version 2021 R1), to generate high-quality thermodynamic distribution maps with various flow-pattern characteristics, such as direct-jet and large-vortex wake. These maps serve as the ground truth for the colorization task. To simulate the real physical process of photon emission tomography (PET), the thermodynamic images are further converted into three-dimensional volume phantoms for GATE (Geant4 Application for Tomographic Emission) simulation. As an advanced Monte Carlo–based platform, GATE can precisely model the entire chain of radionuclide distribution in the flow field, photon generation and transport, and the final detection process. In this work, a detailed PET system model is established (with its simulation interface and key parameters depicted in Figure 9), and the phantoms are input to perform dynamic photon data acquisition. Following the simulation, the collected photon data are reconstructed using the OSEM algorithm to obtain 128 × 128-pixel grayscale γ-photon flow-field images. To meet the input-quality requirements of the colorization task, these grayscale images are preprocessed using a noise-suppressed super-resolution enhancement model based on convolution and Swin Transformer, proposed by Hui et al. This model enhances image clarity and suppresses reconstruction noise, ultimately yielding high-quality grayscale inputs for the SECN model.

#### 4.1.2. Data Processing Platform

Experiments were conducted in a cloud server environment utilizing high-performance computing resources to ensure reliability and reproducibility. The server’s primary computational unit was an NVIDIA RTX 4090 GPU equipped with 24 GB GDDR6X high-speed graphics memory. This hardware provided the necessary processing power to handle 256 × 256 resolution images and support the extensive parameter set of the SECN model, ensuring stable and efficient training.

Regarding the software environment, the experiments were built using the Python 3.8+ programming language. The PyTorch (version 2.5.1+cu121) deep learning framework served as the core computational engine, integrated with the FastAI framework to streamline the model training process. This setup facilitated efficient data processing, model training, and callback management. Image processing tasks utilized PIL (Pillow) and OpenCV libraries for data preprocessing and postprocessing. Scientific computations relied on NumPy and SciPy for numerical calculations and statistical analysis. Evaluation metrics were implemented using scikit-image to ensure accurate and consistent metric calculations. The entire environment supported GPU-accelerated computing with CUDA 11.8+ driver support, significantly enhancing model training efficiency.

### 4.2. Dataset

The original dataset comprised high-quality ANSYS simulation images of the engine exhaust nozzle. Given the scarcity of flow-field data, training deep generative models such as GANs could be prone to overfitting. To bridge the gap between these idealized CFD simulations and the stochastic characteristics of real γ-photon tomography, a multi-level data augmentation strategy was meticulously designed to replicate the major physical disturbances encountered during photon acquisition and image reconstruction. Adjustments to brightness and contrast simulated fluctuations in coincidence count rates arising from variations in radionuclide activity, detector efficiency, and acquisition conditions. Blurring and sharpening operations emulated resolution degradation and high-frequency artifacts caused by finite time-of-flight resolution, angular uncertainty of LORs, and the statistical approximation nature of the OSEM algorithm. Geometric transformations, including cropping, flipping, and small-angle rotation, account for variations in detector orientation and field-of-view positioning across different industrial monitoring setups. Noise injection directly reproduced the Poisson noise and scatter contributions inherent in low-count coincidence event acquisition. By incorporating these physically grounded disturbances, the augmentation strategy substantially enhanced the model’s robustness and generalization capability to real-world γ-photon flow-field imaging conditions.

**Figure 10:**
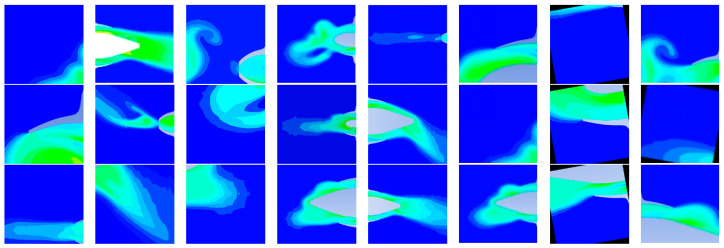
Selected Training Set Images.

**Figure 11:**
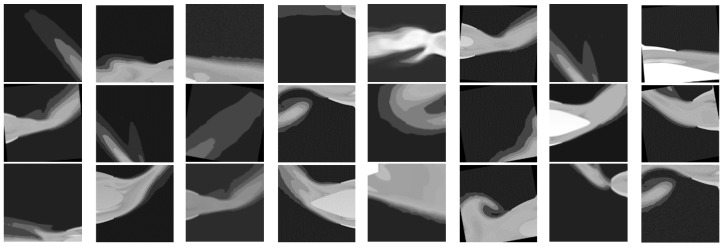
Selected Validation Set Images.

After augmentation, the dataset expanded to 3368 images, which were split into 2422 training images and 946 validation images (approximately a 7:3 ratio). The training also leveraged large-scale datasets such as COCO and ImageNet to increase data diversity and improve robustness. Examples from the training and validation sets are shown in Figure 10 and Figure 11. All images were resized to 256 × 256 pixels, stored in PNG format to preserve quality, and normalized using ImageNet statistics to standardize the inputs. The dataset used single-channel grayscale images as inputs and three-channel RGB images as outputs, directly matching the practical requirements of aero-engine flow-field visualization.

### 4.3. Model Training and Results Analysis

#### 4.3.1. Model Parameter Settings

This study adopts a two-step training strategy, encompassing pre-training and adversarial training phases.

During the pre-training phase, which spans 20 epochs, the optimization objective is solely based on the perceptual loss (Content Loss) derived from the VGG network, without the involvement of a discriminator. This approach enables the generator to acquire the fundamental mapping from grayscale to color images and to grasp the overall structural and textural details of the images before being exposed to adversarial signals. It provides a stable and high-quality starting point for subsequent adversarial training, effectively mitigating the pattern collapse issues commonly encountered in later adversarial stages.

The adversarial training phase is refined using the WGAN-GP (Wasserstein GAN with Gradient Penalty) framework. WGAN-GP utilizes Wasserstein distance and gradient penalty to enforce the Lipschitz constraint [26]. The update ratio between the discriminator and generator is set to 5:1, implying that the generator undergoes a single update for every five updates of the discriminator. This ensures that the discriminator consistently delivers accurate and reliable gradient signals to the generator. Additionally, a cosine annealing learning rate scheduling strategy is implemented during training, smoothly reducing the learning rate from ${5\;\times \;10}^{-5}$ to ${5\;\times \;10}^{-6}$. Detailed model training parameters and hyperparameter configurations are summarized in Table 1.

#### 4.3.2. Evaluation Metrics

**Figure 12:**
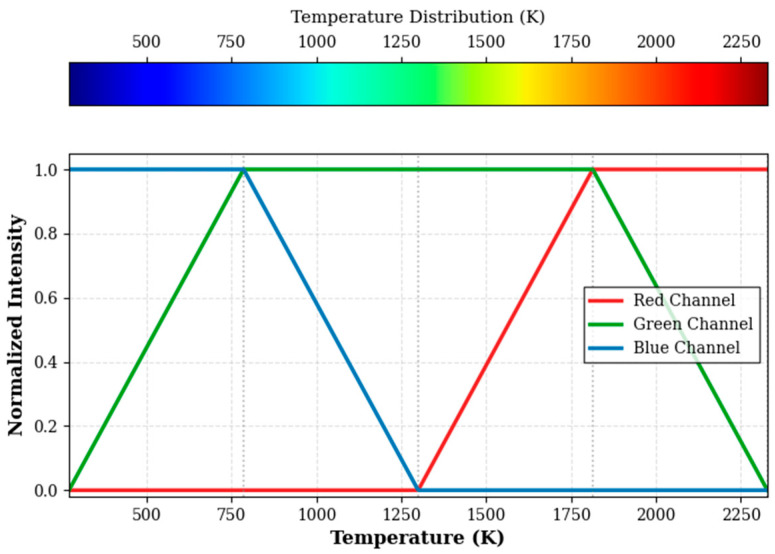
RGB–temperature Mapping Relationship.

To comprehensively evaluate the final performance of the SECN model, a deterministic mapping model between the RGB color space and the flow-field temperature (K) is established, based on the standard thermodynamic colormap utilized in ANSYS CFD simulations. As depicted in Figure 12, this mapping is defined as a piecewise linear function, characterized by five key anchor points: 271.3 K, 785.5 K, 1299.7 K, 1813.8 K, and 2328.0 K.

The normalized intensities of the red (R), green (G), and blue (B) channels exhibit linear variations across different temperature intervals, with each channel alternately taking dominance. For instance, within the temperature range of 785.5 K to 1299.7 K, the blue-channel intensity decreases linearly while the green-channel intensity increases linearly. This channel-crossing behavior reflects complex mixing transition regions within the flow field. Utilizing this mapping model, generated pseudo-color images can be inversely transformed back to the temperature field. The APE between the inverted temperatures in gradient-jump regions and the CFD ground truth can then be calculated to quantitatively evaluate SECN’s capability in preserving flow-field structural integrity. The APE is defined as shown in Formula (18):

(18) $$\mathrm{APE}=\left|\frac{T_{Pred}(i)-T_{GT}(i)}{T_{GT}(i)}\right|\times 100\%,$$

where $T_{GT}(i)$ denotes the CFD ground-truth temperature at the *i*-th pixel (in K), and $T_{\mathrm{Pr}ed}(i)$ is the predicted temperature inversely derived from the colorized image using the established RGB–temperature mapping function. To provide an overall error level, the mean absolute percentage error (MAPE) is computed as shown in Formula (19):

(19) $$\mathrm{MAPE}=\frac{1}{N}\sum_{i=1}^{N}\mathrm{APE},$$

Peak Signal-to-Noise Ratio (PSNR) serves as a critical metric for assessing image reconstruction quality, calculated as shown in Formula (20):

(20) $$\mathrm{PSNR}=20\times \log_{10}\left(\frac{{\mathrm{MAX}}_{I}}{\sqrt{\mathrm{MSE}}}\right),$$

where MSE denotes the Mean Squared Error, calculated as shown in Formula (21):

(21) $$\mathrm{MSE}=\frac{1}{M\times N}\times \sum_{i=1}^{M}\sum_{j=1}^{N}{\left(I_{i,j}-K_{i,j}\right)}^{2},$$

where *M* and *N* denote the number of rows and columns in the image, respectively. $I_{i,j}$ represents the pixel value of the original image, $K_{i,j}$ denotes the pixel value of the reconstructed image, and ${\mathrm{MAX}}_{I}$ is the maximum value of the image pixels (typically 1.0). A higher PSNR value indicates greater similarity between the reconstructed image and the original image, signifying superior image quality.

The Structural Similarity (SSIM) Index measures the SSIM between images, as shown in Formula (22):

(22) $$\mathrm{SSIM}=\frac{\left(2\mu_{x}\mu_{y}+C_{1})(2\sigma_{xy}+C_{2}\right)}{\left(\mu_{x}^{2}+\mu_{y}^{2}+C_{1})(\sigma_{x}^{2}+\sigma_{y}^{2}+C_{2}\right)},$$

where $\mu_{x}$ and $\mu_{y}$ are the respective means of the two images, $\sigma_{x}^{2}$ and $\sigma_{y}^{2}$ are the respective variances, $\sigma_{xy}$ is the covariance, and C_1_, C_2_ are constant terms. SSIM values range between [−1, 1], with values closer to 1 indicating higher SSIM.

Mean Absolute Error (MAE) measures pixel-level error, as shown in Formula (23):

(23) $$\mathrm{MAE}=\frac{1}{N}\times \sum_{i=1}^{N}|y_{i}-{\hat{y}}_{i}|,$$

where *N* is the total number of pixels, $y_{i}$ is the true pixel value, and ${\hat{y}}_{i}$ is the predicted pixel value. A smaller MAE value indicates higher pixel-level accuracy.

The Fréchet Inception Distance (FID) measures the realism and quality of generated images, as shown in Formula (24):

(24) $$\mathrm{FID}=||\mu_{r}-\mu_{g}|{|}^{2}+\mathrm{Tr}(\Sigma_{r}+\Sigma_{g}-2{\left(\Sigma_{r}\Sigma_{g}\right)}^{1/2}),$$

where $\mu_{r}$ and $\mu_{g}$ represent the mean feature values of the real and generated images, respectively, and $\Sigma_{r}$ and $\Sigma_{g}$ denote the corresponding covariance matrices. A smaller FID value indicates that the generated image distribution is closer to that of the real images.

Additionally, to further quantify the information fidelity of the colorized images, Shannon entropy is introduced as an additional evaluation metric. For each colorized image, the entropy of the three RGB channels is calculated based on the probability distribution of pixel intensities, and the average value is taken as the overall image entropy H, as shown in Formula (25):

(25) $$\mathrm{Entropy}=-\sum_{i}p_{i}\log_{2}p_{i},$$

For each channel, the pixel intensity values are first quantized into discrete bins (e.g., 0–255), and the frequency of each bin is divided by the total number of pixels to obtain $p_{i}$. This normalization ensures that $\sum_{i}p_{i}=1$, where $p_{i}$ reflects the likelihood of observing a specific pixel intensity in the image. A higher entropy value indicates a more diverse distribution of pixel intensities, meaning that the image contains richer information content and a more detailed structure.

### 4.4. Algorithms Performance Comparison

To thoroughly evaluate the effectiveness of the proposed algorithm, comparative experiments were conducted against leading approaches. The comparison methods include the classic DeOldify algorithm and the latest DDColor dual-decoder coloring network, representing traditional and state-of-the-art applications in image coloring.

#### 4.4.1. Analysis of the Model Training Process

**Figure 13:**
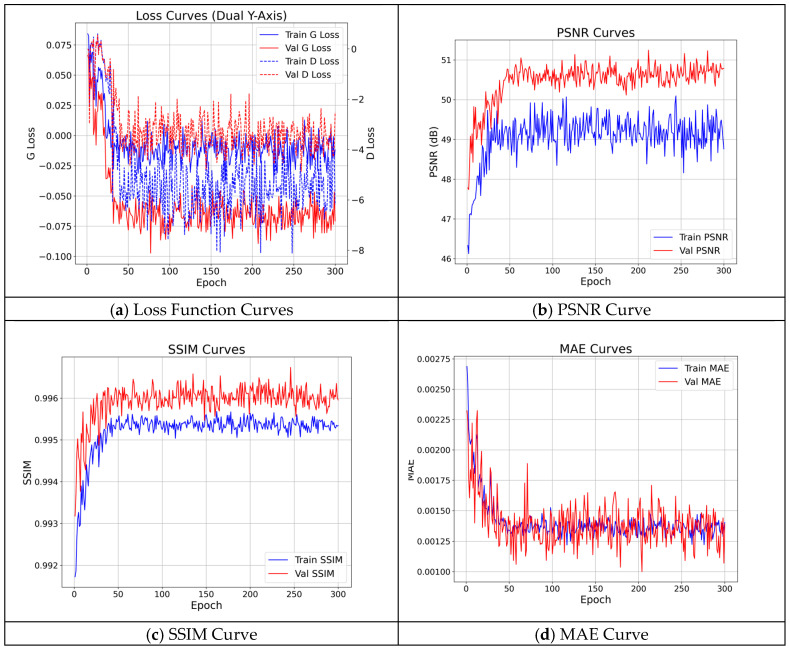
Evolution of Loss Functions and PSNR During Training.

To evaluate the model’s convergence and generalization ability, key performance metrics of the SECN model were recorded and visualized throughout training, as shown in Figure 13. This figure illustrates the changes in the generator and discriminator loss functions, PSNR, SSIM, and MAE across the training and validation sets as epochs progress.

As depicted in Figure 13, during the initial training phase, the generator loss (G Loss) demonstrates a rapid decline followed by a gradual stabilization, ultimately reaching a dynamic equilibrium. This trend indicates that the generator has effectively acquired the complex mapping from input to output. The oscillation observed in the discriminator loss (D Loss) reflects the adversarial nature inherent in a GAN network, where the generator and discriminator alternately enhance their capabilities. This characteristic oscillation is typical of GAN training convergence and suggests robust generalization ability. Simultaneously, the PSNR and SSIM metrics on the validation subset show steady improvement with increasing training iterations, while the MAE metric consistently decreases, eventually reaching a stable point. This alignment with the convergence trend of the loss functions intuitively demonstrates that the quality of images generated by the model is continuously enhanced and ultimately converges to a high standard. Notably, the peaks of the PSNR and SSIM curves occur around the 212th iteration, coinciding with the lowest error value on the MAE curve. Consequently, this paper adopts the weight files obtained at this iteration as the final training results.

#### 4.4.2. Qualitative Analysis

**Figure 14:**
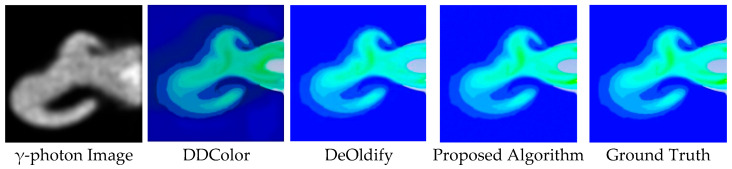
Colorization Results for the Large-scale Vortex Wake Case.

**Figure 15:**
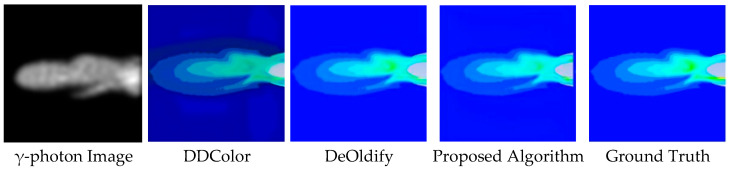
Colorization Results for the Horizontal Wake Case.

In terms of color banding artifacts, the DDColor algorithm exhibits noticeable discontinuities in gradient transitions within the engine nozzle region, characterized by unnatural color jumps between high- and medium-temperature areas, lacking smooth transitions. Similarly, the DeOldify algorithm suffers from gradient discontinuity, producing abrupt color changes at the jet boundary, as illustrated in Figure 14 and Figure 15. In contrast, when compared to the ground-truth image, the proposed algorithm demonstrates superior continuity and structural rationality in suppressing color banding artifacts. Specifically, the transition from yellow–green tones in the high-temperature core to blue tones toward the edges is smoother and more natural, avoiding unreasonable jumps and better aligning with the temperature-field distribution within the engine nozzle.

#### 4.4.3. Quantitative Results Comparison

To conduct a thorough evaluation of the final performance of the proposed algorithm based on the SECN model, this paper uses the best-performing model saved during training to conduct inference on a validation set comprising 946 images, calculating the average across all evaluation metrics. For a fair comparison, all contrastive algorithms underwent identical training and testing procedures on the same dataset. The experimental results demonstrate that the proposed algorithm achieves superior performance across all metrics, as detailed in Table 2, ↑ indicates higher is better, ↓ indicates lower is better. Compared to the DeOldify and DDColor algorithms, this approach shows improvements in the FID, PSNR, SSIM, and MAE metrics. In addition, Shannon entropy was calculated to quantify the information content of the colorized images, with average values of 3.1574 for DDColor, 3.8543 for DeOldify, and 4.0257 for the proposed algorithm. These results indicate that SECN not only delivers superior visual quality but also retains more structural details and richer information content, thereby enhancing reliability for downstream temperature inversion tasks. For precision-oriented tasks like flow field inversion, even minor improvements in data quality can effectively prevent erroneous judgments about physical states. The color restoration results produced by our algorithm are closer to real images, with more accurate shading, particularly when handling images with complex geometric structures and textures, such as engine nozzles, where the proposed algorithm exhibits stronger adaptability.

#### 4.4.4. Comparison of Temperature Inversion Results

**Figure 16:**
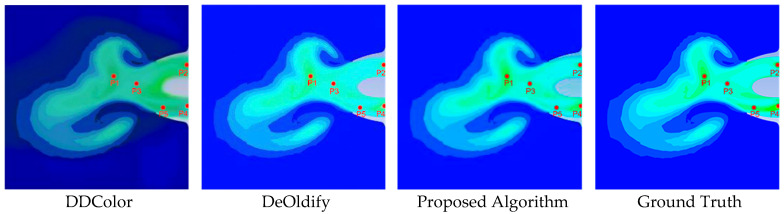
Sampling Points for Temperature Inversion.

To verify the accuracy of color restoration, a fixed-point sampling strategy was adopted for the temperature inversion experiment. As illustrated in Figure 16, five points (P1–P5) were selected to represent key flow-field regions.

Based on the selected points, a localized entropy analysis was first conducted to comprehensively evaluate the preservation of structural gradients and the suppression of color banding artifacts. Specifically, 31 × 31 pixel patches centered at each coordinate were extracted for analysis.

As shown in Table 3, the Ground Truth inherently exhibits low local entropy, reflecting the smooth and continuous nature of the γ-photon flow field gradients. In contrast, traditional methods such as DeOldify and DDColor produce severe color banding artifacts in these transition regions. These artifacts artificially scatter the pixel distribution, leading to an abnormal increase in local entropy. The proposed SECN model effectively mitigates these staircase-like discontinuities through its structure-aware enhancement module, maintaining structurally continuous gradients and preventing artificial entropy inflation. Consequently, the localized entropy values generated by the SECN model most closely approximate those of Ground Truth.

The RGB values at these coordinates were extracted from the Ground Truth and images generated by the proposed algorithm, DeOldify, and DDColor. Using the established RGB–temperature mapping shown in Figure 12, the (R,G,B) values were inversely mapped to temperature readings (in K). The detailed quantitative comparison is listed in Table 4.

Crucially, the preservation of localized structural information directly influences the accuracy of downstream physical quantitative analysis. As detailed in Table 4, the transition regions where baseline methods exhibit inflated local entropy correspond precisely to significant temperature inversion errors. In contrast, SECN maintains high structural fidelity at the micro-level, achieving a significantly reduced overall temperature inversion MAPE of 7.60%.

The experimental results demonstrate that the proposed algorithm, based on the SECN model, achieves high accuracy in both structural and color restoration. In particular, at P4—a transition region prone to color artifacts—DeOldify and DDColor fail to correctly identify the region and erroneously fill it with cool tones. In contrast, the temperature inverted by the proposed algorithm closely matches the ground truth with only a minor deviation, underscoring its superior performance.

### 4.5. Ablation Studies

**Figure 17:**
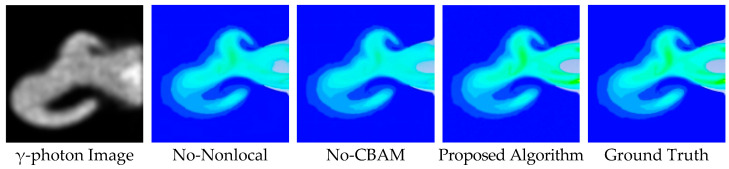
Qualitative Comparison of Ablation Experiments.

To verify the effectiveness of the proposed components, ablation experiments were conducted on the key modules within the SAE module. The objective of this ablation study was to quantify the contribution of each component to the overall performance and validate the rationality of the design. The experimental setup included three configurations: the full model, the model without the CBAM attention module (No-CBAM), and the model without the NonlocalBlock (No-Nonlocal). This design effectively demonstrated the critical roles of CBAM and NonlocalBlock in the engine exhaust-nozzle image colorization task. As illustrated in Figure 17, removing CBAM resulted in spatial inconsistency in the nozzle region and failed to accurately recover the colors of high-temperature areas, indicating that CBAM is essential for guiding the network to focus on key semantic regions. The No-Nonlocal configuration produced severe color banding artifacts, leading to obvious color discontinuities at the boundaries between high- and low-temperature regions, suggesting that the network could not effectively propagate global context without the NonlocalBlock. By integrating both modules, the proposed algorithm achieved a natural and continuous gradient distribution.

**Figure 18:**
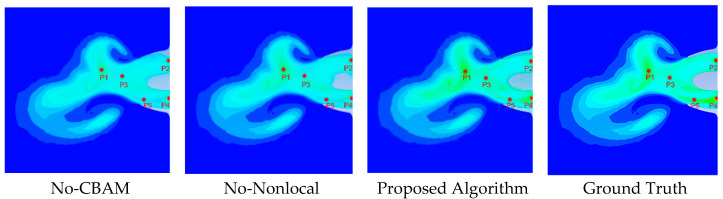
Sampling Points for Temperature Inversion in the Ablation Study.

To explicitly clarify how these modules suppress color banding artifacts, we examined their respective mechanisms and effects on specific regions. The NonLocalBlock computes global attention weights across all positions in the feature map, allowing distant high-temperature core information to propagate to boundary pixels. This long-range dependency modeling effectively mitigated local discontinuities that would otherwise appear as stair-like color jumps, as clearly observed in the high-to-low temperature transition zones of Figure 17. In parallel, the CBAM applied channel attention to emphasize semantically important features and spatial attention to highlight jet boundaries and temperature gradient zones. Adaptively recalibrating responses prevented localized receptive fields from misinterpreting gradual probability gradients as abrupt semantic boundaries. These synergistic effects were quantitatively validated in specific gradient-transition sampling regions (P1–P5 in Figure 18 and Table 5). For instance, at point P4—a sensitive transition boundary highly prone to banding artifacts—the absence of CBAM or NonLocalBlock led to severe temperature inversion errors of 702.75 K and 700.69 K, respectively. In contrast, the full model dynamically resolved this gradient ambiguity, reducing the error at P4 to 4.11%, and bringing the overall MAPE down from 25.65% (No-CBAM) and 24.01% (No-Nonlocal) to 7.60%. The complementary action of NonLocalBlock and CBAM thus restored smooth physical gradients and eliminated the banding phenomenon observed in the ablated models.

Shannon entropy analysis further revealed that the average entropy values were 3.5926 for No-CBAM, 3.7576 for No-Nonlocal, and 4.0257 for the proposed algorithm, confirming that both CBAM and NonlocalBlock contribute to increased information content and optimal structural detail preservation. The synergy between CBAM and NonlocalBlock effectively suppressed color banding artifacts and yielded smoother, structurally coherent transitions.

Quantitative analysis of the ablation experiments is shown in Table 5, ↑ indicates higher is better, ↓ indicates lower is better.

Temperature inversion analysis was further conducted for the ablation models, and the sampling points for the inversion experiment are shown in Figure 18.

The quantitative metrics in Table 5 indicate that removing either the CBAM or NonLocalBlock reduces overall information fidelity. This observation aligns with the point-anchored local entropy analysis at the five key sampling points displayed in Figure 18. Without the complete structure-aware enhancement module, the ablated models failed to suppress staircase-like discontinuities in transition regions, leading to an abnormal inflation of local entropy. Specifically, across these five points, the average local entropy rose from 4.4836 in the proposed SECN to 4.5950 for No-CBAM and 4.6848 for No-Nonlocal. This loss of structural continuity at the micro level directly explained the increased temperature inversion errors observed in the ablated models in Table 6.

The temperature inversion results indicate that both CBAM and NonlocalBlock are indispensable components. CBAM ensures that the high-temperature core region is correctly identified, while NonlocalBlock is crucial for maintaining continuous color gradients.

## 5. Conclusions

This study tackles the prevalent issue of color banding artifacts in γ-photon flow-field image colorization and introduces a novel colorization algorithm based on the SECN model. The proposed algorithm leverages a hybrid U-Net and GAN framework, with both the generator and discriminator incorporating the SAE module. This integration aims to bolster structural integrity and mitigate high-frequency artifacts stemming from probabilistic imaging noise. Within SAE, a four-branch multi-scale dilated convolutional network is employed to capture flow-field features across varying receptive fields. Subsequently, the NonlocalBlock and CBAM are integrated to establish a multi-attention mechanism that adaptively highlights semantically significant regions, such as the high-temperature core, thereby ensuring smooth gradient transitions and rich texture details in the generated results. Experimental results underscore the algorithm’s robust performance in γ-photon flow-field inversion-related evaluation, obtaining PSNR, SSIM, FID, and MAE values of 32.5831, 0.8612, 17.8514, and 0.0191, respectively, corresponding to improvements over DeOldify of 4.54%, 2.82%, 5.18%, and 11.16%. From the perspective of information entropy, SECN yields an average entropy value of 4.0257, a 4.44% enhancement over DeOldify’s 3.8543. This higher entropy reflects superior preservation of information richness and reduced uncertainty in reconstructing complex probabilistic structures, aligning with information-theoretic principles in scientific imaging. Ablation studies further validate the pivotal roles of multi-scale dilated convolutions, CBAM attention, and the NonLocalBlock mechanism. In temperature inversion experiments, the proposed algorithm achieves a MAPE of 7.60%, a significant reduction of 18.42 percentage points from DeOldify’s 26.02%, indicating effective correction of semantic loss in high-temperature regions and improved gradient continuity. Despite these advancements, SECN still exhibits color bleeding near certain boundaries. Future research will focus on integrating the Transformer with the U-Net to enhance global context modeling and improve boundary consistency.

## Figures and Tables

**Table 1:** Model Training Parameters and Hyperparameter Configuration.

Parameters	Value
Batch Size	4
Network Factor	2
Optimizer	Adam
Initial Learning Rate	5 × 10^−5^
Final Learning Rate	5 × 10^−6^
Optimizer Parameters (Betas)	(0.0, 0.9)
Training Epochs	100
D/G Training Ratio	5:1

**Table 2:** Quantitative Analysis of Shading Effects.

	FID ↓	PSNR ↑	SSIM ↑	MAE ↓	Entropy ↑
DeOldify	18.8261	31.1684	0.8376	0.0215	3.8543
DDColor	49.6979	14.0462	0.6219	0.1369	3.1574
Proposed algorithm	17.8541	32.5831	0.8612	0.0191	4.0257

**Table 3:** Local Patch Entropy at Temperature Sampling Points.

	GT Entropy	DeOldify Entropy	DDcolor Entropy	Proposed Algorithm Entropy
P1	1.5786	4.4173	4.7982	3.7273
P2	2.1508	4.9171	4.8255	4.7392
P3	0.6884	3.4731	3.9843	2.9620
P4	3.6888	5.8585	5.8409	5.7779
P5	2.8827	5.2601	5.5161	5.2114
Avg.	2.1979	4.7852	4.9930	4.4836

**Table 4:** Quantitative Analysis of Temperature Inversion.

	GT(R,G,B)	GT Temp	DeOldify Temp	DDcolor Temp	Proposed Algorithm Temp	Proposed Algorithm APE
P1	(0,255,101)	1095.91	1584.12	1055.57	1132.14	3.31%
P2	(0,255,100)	1097.97	936.59	1079.86	995.05	9.37%
P3	(0,255,208)	880.19	870.31	971.17	904.98	2.82%
P4	(210,255,0)	1723.25	862.07	951.00	1652.45	4.11%
P5	(0,255,118)	1061.74	852.19	1063.80	866.19	18.42%
MAPE	-	-	26.02%	12.13%	7.60%	7.60%

**Table 5:** Quantitative Analysis of Ablation Experiments.

	FID ↓	PSNR ↑	SSIM ↑	MAE ↓	Entropy ↑
No-Nonlocal	18.7269	21.0306	0.8004	0.0616	3.7576
No-CBAM	18.8133	20.8247	0.7869	0.0645	3.5926
Proposed algorithm	17.8541	32.5831	0.8612	0.0191	4.0257

**Table 6:** Quantitative Analysis of Temperature Inversion in the Ablation Study.

	GT(R,G,B)	GT Temp	No-CBAM Temp	No_Nonlocal Temp	Proposed Algorithm Temp	Proposed Algorithm APE
P1	(0,255,101)	1095.91	942.77	992.99	1132.14	3.31%
P2	(0,255,100)	1097.97	896.24	914.36	995.05	9.37%
P3	(0,255,208)	880.19	819.67	839.84	904.98	2.82%
P4	(210,255,0)	1723.25	702.75	700.69	1652.45	4.11%
P5	(0,255,118)	1061.74	745.15	743.10	866.19	18.42%
MAPE	-	-	25.65%	24.01%	7.60%	7.60%

## Data Availability

The γ-photon flow-field images used in the study come from ANSYS FLUENT and GATE simulation experiments. The datasets used and/or analyzed in the current study are available from the corresponding author on reasonable request. Our team has released the code as open source and uploaded it to a public code hosting platform. The project repository is available at: https://github.com/holy620/SECN-model (accessed on 14 February 2026). Correspondence and requests for materials could be addressed to Xiao Hui.

## References

[1] Hampel U., Bieberle A., Hoppe D., Kronenberg J., Schleicher E., Sühnel T., Zimmermann F., Zippe C. (2007). High resolution gamma ray tomography scanner for flow measurement and non-destructive testing applications. Rev. Sci. Instrum..

[2] Bruggemann J., Gross A., Pate S. (2020). Non-intrusive visualization of optically inaccessible flow fields utilizing positron emission tomography. Aerospace.

[3] Stewart C.V. (1999). Robust parameter estimation in computer vision. SIAM Rev..

[4] Oliverio T.N., Prasetyo S.Y. (2024). Color and attention for U: Modified multi attention U-Net for a better image colorization. JOIV Int. J. Inform. Vis..

[5] Schlemper J., Oktay O., Schaap M., Heinrich M., Kainz B., Glocker B., Rueckert D. (2019). Attention gated networks: Learning to leverage salient regions in medical images. Med. Image Anal..

[6] Shannon C.E. (1948). A mathematical theory of communication. Bell Syst. Tech. J..

[7] Wang Z., Zhuang J., Ye S., Xu N., Xiao J., Peng C. (2023). Image restoration quality assessment based on regional differential information entropy. Entropy.

[8] Ke Z., Zheng W., Wang X., Lin M. (2024). Information entropy analysis of a PIV image based on wavelet decomposition and reconstruction. Entropy.

[9] He Y., Xiao L. (2024). Structured pruning for deep convolutional neural networks: A survey. IEEE Trans. Pattern Anal. Mach. Intell..

[10] Shorten C., Khoshgoftaar T.M. (2019). A survey on image data augmentation for deep learning. J. Big Data.

[11] Liu X., Zhang F., Hou Z., Mian L., Wang Z., Zhang J., Tang J. (2023). Self-supervised learning: Generative or contrastive. IEEE Trans. Knowl. Data Eng..

[12] Yamashita R., Nishio M., Do R.K.G., Togashi K. (2018). Convolutional neural networks: An overview and application in radiology. Insights Imaging.

[13] Khan S.H., Naseer M., Hayat M., Zamir S.W., Khan F.S., Shah M. (2021). Transformers in vision: A survey. ACM Comput. Surv..

[14] Song X., Chao H., Xu X., Guo H., Xu S., Turkbey B., Wood B.J., Sanford T., Wang G., Yan P. (2022). Cross-modal attention for multi-modal image registration. Med. Image Anal..

[15] Wang W., Tan X., Zhang P., Wang X. (2022). A CBAM based multiscale transformer fusion approach for remote sensing image change detection. IEEE J. Sel. Top. Appl. Earth Obs. Remote Sens..

[16] Ronneberger O., Fischer P., Brox T. U-Net: Convolutional networks for biomedical image segmentation. Proceedings of the International Conference on Medical Image Computing and Computer-Assisted Intervention (MICCAI).

[17] Wang Z. (2023). Research on Image Colorization Algorithms Based on Classification Loss Functions. Master’s Thesis.

[18] Kang X., Yang T., Ouyang W., Ren P., Li L., Xie X. DDColor: Towards photo-realistic image colorization via dual decoders. Proceedings of the IEEE/CVF International Conference on Computer Vision(ICCV).

[19] Goodfellow I., Pouget-Abadie J., Mirza M., Xu B., Warde-Farley D., Ozair S., Courville A., Bengio Y. Generative adversarial nets. Proceedings of the Advances in Neural Information Processing Systems (NIPS).

[20] Isola P., Zhu J.-Y., Zhou T., Efros A.A. Image-to-image translation with conditional adversarial networks. Proceedings of the IEEE Conference on Computer Vision and Pattern Recognition (CVPR).

[21] Wang T.-C., Liu M.-Y., Zhu J.-Y., Tao A., Kautz J., Catanzaro B. High-resolution image synthesis and semantic manipulation with conditional GANs. Proceedings of the IEEE Conference on Computer Vision and Pattern Recognition.

[22] Vitoria P., Raad Cisa L., Ballester C. ChromaGAN: Adversarial picture colorization with semantic class distribution. Proceedings of the IEEE/CVF Winter Conference on Applications of Computer Vision (WACV).

[23] Hospedales T., Antoniou A., Micaelli P., Storkey A. (2022). Meta-learning in neural networks: A Survey. IEEE Trans. Pattern Anal. Mach. Intell..

[24] Kim Y., Cho Y., Nguyen T.-T., Hong S., Lee D. MetaWeather: Few-Shot weather-degraded image restoration. Proceedings of the European Conference on Computer Vision (ECCV).

[25] Zhai X., Oliver A., Kolesnikov A., Beyer L. S4L: Self-supervised semi-supervised learning. Proceedings of the IEEE/CVF International Conference on Computer Vision (ICCV).

[26] Xiao H., Liu Q., Xu Y., Wang M., Liu J. (2025). Research on a noise-suppression super-resolution enhancement module for positron flow field images based on convolution and SwinTransformer structures. Sci. Rep..

[27] Baik S., Choi J., Kim H., Cho D., Min J., Lee K.M. Meta-learning with task-adaptive loss function for few-shot learning. Proceedings of the IEEE/CVF International Conference on Computer Vision (ICCV).

[28] Woo S., Park J., Lee J.-Y., Kweon I.S. CBAM: Convolutional block attention module. Proceedings of the European Conference on Computer Vision (ECCV).

[29] Gulrajani I., Ahmed F., Arjovsky M., Dumoulin V., Courville A. Improved training of Wasserstein GANs. Proceedings of the Advances in Neural Information Processing Systems (NIPS).

